# Surface-Condition-Dependent Deformation Mechanisms in Lead Nanocrystals

**DOI:** 10.34133/2022/9834636

**Published:** 2022-07-27

**Authors:** Hongtao Zhang, Wen Wang, Jun Sun, Li Zhong, Longbing He, Litao Sun

**Affiliations:** ^1^SEU-FEI Nano-Pico Center, Key Laboratory of MEMS of Ministry of Education, Southeast University, Nanjing 210096, China; ^2^Center for Advanced Materials and Manufacture, Southeast University-Monash University Joint Research Institute, Suzhou 215123, China

## Abstract

Serving as nanoelectrodes or frame units, small-volume metals may critically affect the performance and reliability of nanodevices, especially with feature sizes down to the nanometer scale. Small-volume metals usually behave extraordinarily in comparison with their bulk counterparts, but the knowledge of how their sizes and surfaces give rise to their extraordinary properties is currently insufficient. In this study, we investigate the influence of surface conditions on mechanical behaviors in nanometer-sized Pb crystals by performing *in situ* mechanical deformation tests inside an aberration-corrected transmission electron microscope (TEM). Pseudoelastic deformation and plastic deformation processes were observed at atomic precision during deformation of pristine and surface-oxidized Pb particles, respectively. It is found that in most of the pristine Pb particles, surface atom diffusion dominates and leads to a pseudoelastic deformation behavior. In stark contrast, in surface-passivated Pb particles where surface atom diffusion is largely inhibited, deformation proceeds via displacive plasticity including dislocations, stacking faults, and twinning, leading to dominant plastic deformation without any pseudoelasticity. This research directly reveals the dramatic impact of surface conditions on the deformation mechanisms and mechanical behaviors of metallic nanocrystals, which provides significant implications for property tuning of the critical components in advanced nanodevices.

## 1. Introduction

As the feature size of nanodevices gradually decreases to the sub-10 nm scale, surface conditions have been proved to dominate properties and behaviors of nanomaterials [1–3]. Particularly, surface-mediated mechanical deformation process of metals has attracted interest since metallic materials are usually affected by external stress in practical applications [4–6]. Previous studies have shown that many factors, including temperature and material size, may affect and determine the deformation process of materials [4, 5, 7–12]. Based on dislocation dynamics which is common and dominant for bulks, the traditional theories have been successfully applied to explain deformation processes of many metals such as Au, W, Pt, Bi, and Sn [9, 11–15]. Moreover, recently, it was also found that as material size decreases to nanometer scale, surface diffusion would predominantly affect the deformation process of metals [12, 16, 17], which successfully explains several unusual mechanical deformation phenomena observed in metallic nanocrystals [7, 12, 16, 18].

The mechanical deformation processes of different small-volume metals have been extensively studied. Most previous studies pursued the intrinsic mechanical properties of small-volume metals under ideal conditions where clean sample surfaces were obtained by either using inert metals such as Au and Pt [8, 10, 14, 15, 19, 20] or adopting techniques including plasma cleaning, electron beam etching, and *in situ* welding [2, 16, 18, 21, 22]. In practical applications, however, devices often have complex surfaces that are oxidized or corroded after long-time exposure to their serving environments, e.g., in air or liquids [1, 3, 6, 23–26]. Thus, the impact of surface conditions on deformation of small-volume metals should not be neglected. Indeed, some studies have already shown that surface coating remarkably affects the sintering of metals [2, 17]. Yet the effect of surface passivation on the mechanical behavior of metals and the mechanisms involved therein remains largely unknown. Thus, it is necessary to provide direct and real-time observation of the deformation processes of metallic materials with different surface conditions at atomic precision.

Lead is one of the earliest metals that have wide technological applications in many fields such as alloys, glasses, lead-acid batteries, or photovoltaic devices [27–30]. The mechanical properties of Pb under distinct conditions have also been studied since the deformation process of Pb can significantly influence the performances of devices [5, 6, 31]. Nevertheless, there is a lack of direct evidence on the deformation of Pb nanoparticles, and details of the surface-mediated deformation process have not yet been fully clarified.

In this work, *in situ* mechanical deformation experiments were conducted on Pb particles with distinct surface conditions in an aberration-corrected Titan TEM/STEM. Surface-condition-dependent pseudoelastic deformation and plastic deformation were observed. In pure Pb nanoparticles, the prevailing surface diffusion process contributes to pseudoelastic deformation, while in passivated Pb particles with a PbO surface layer, surface diffusion is prohibited and gives way to crystal slips such as dislocations, stacking faults, and twinning, leading to irreversible plastic deformation.

## 2. Results and Discussion

Preparation of Pb particles with distinct surface conditions is shown in Materials and Methods. Typical results are shown in Figure S1 and Figure S2. More details can be found in our previous research [32].

**Figure 1:**
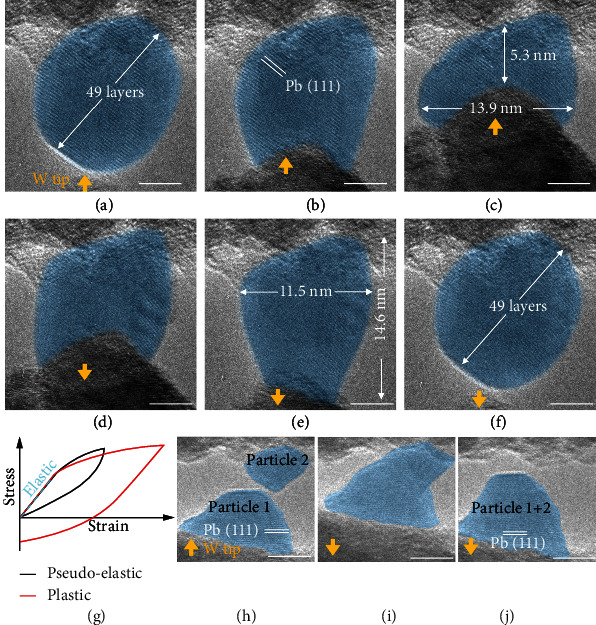
Surface diffusion inducing (a–g) liquid-like pseudoelastic deformation and (h–j) coalescence of pure Pb particles. (a) Initial morphology of Pb particle before deformation. (b–e) Shape evolution of the Pb particle during the deformation process. (f) Final morphology of the Pb particle after deformation. (g) Illustration of plastic and pseudoelastic deformation. (h) Initial morphology of 2 Pb particles before coalescence. (i) Coalescence process of particles. (j) Final morphology of Pb particle after coalescence. For better view, all Pb particles have been painted blue. All the orange arrows indicate the movement direction of W tip. Scale bars: 5 nm.

*In situ* mechanical deformation tests were conducted on pure Pb particles with clean surfaces, and the liquid-like pseudoelastic deformation process has been captured, as shown in Figure 1 and Figure S3. Initially, a Pb particle supported on the PX-PbTiO_3_ nanowire surface was relaxed nearly into an ellipsoid, where 49 layers of Pb(111) planes can be identified, as shown in Figure 1(a). The two diameters of the ellipsoid particle were measured to be 11.7 nm and 12.9 nm, respectively. Then, a W tip was moved upward to press the Pb particle in Figure 1(b). As the W tip kept moving upward, the particle was gradually compressed into a thick slice in Figure 1(c) and Figure S3(d). The width increased to 13.9 nm and thickness decreased to 5.3 nm. Then, the W tip was moved downward away from the nanowire, and the Pb particle was relaxed in Figure 1(d) and was even slightly stretched in Figure 1(e) due to the van der Waals forces. As the W tip still moved downward, the van der Waals forces became too weak and the Pb particle lost its contact with the tip. Meanwhile as shown in Figure 1(f), the Pb particle quickly shrank into an ellipsoid which was almost the same with its initial shape in Figure 1(a). Moreover, the crystal fringes of Pb{111} can be observed clearly during the whole process, and this proved that the particle is in a solid state. These results clearly represent pseudoelastic deformation instead of the common plastic deformation process, as illustrated in Figure 1(g).

A similar pseudoelastic deformation phenomenon has been observed in sub-10 nm Ag particles in a previous study, and it was proved to be induced by the diffusion of surface atoms [16]. It has been shown that such recoverable shape change is mediated by diffusion of surface atoms and driven by the minimization of surface energy. In fact, surface diffusion can lead to other phenomena. As shown in Figures 1(h)–1(j) for instance, coalescence of two pure Pb particles during mutual extrusion was observed. The two particles were independently placed on a W tip and substrate in Figure 1(h), respectively. As the tip moved upward, the two particles touched and pressed against each other in Figure 1(i). Then, as the tip moved downward, the initial two particles have merged into one bigger particle in Figure 1(j). Similar diffusion-induced coalescence processes have also been observed in other materials [2, 17].

**Figure 2:**
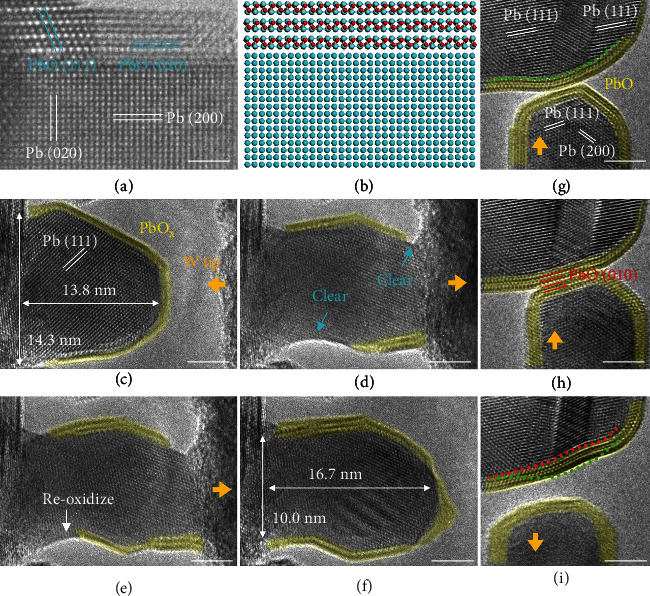
Impact of surface oxidation on the mechanical behavior of Pb particles. (a) Experimental TEM image of one surface-oxidized Pb particle. (b) Model of the surface-oxidized Pb particle based on (a). (c) Initial morphology of Pb particle covered by PbO_x_ before deformation. (d and e) Morphology evolution of the particle during stretching. (f) Final morphology of the particle after deformation. (g) Initial morphology of 2 oxidized Pb particles. (h) Morphology evolution of the particles during deformation. (i) Final morphology of the particles. In (g) and (i), the green and red dotted lines show the initial and final shape of Pb particle. For better view, surface PbO_x_ layers have been painted yellow. All the orange arrows indicate the movement direction of W tip. Scale bars: (a): 2 nm; (c)–(i): 5 nm.

Since surface diffusion plays a vital role during deformation of Pb nanoparticles, the surface condition of Pb particles may significantly affect these diffusive deformation processes. Then, the influence of surface passivation during mechanical deformation process was studied. PbO_x_ layers were introduced to the surface of Pb particles by *in situ* irradiation, and the details can be found in our previous research [32]. Examples are shown in Figures 2(a) and 2(b) and Figure S4. Mechanical deformation experiments were then conducted on surface-passivated Pb particles.

As shown in Figures 2(c)–2(f) and Figure S5, when the surface of Pb nanoparticle is oxidized, plastic deformation instead of the pseudoelastic deformation was observed during the similar *in situ* compression and stretching processes. Initially, a Pb particle supported on a PX-PbTiO_3_ nanowire was completely covered with a PbO_x_ surface layer in Figure 2(c). The particle was measured to be 13.8 nm in height and the 14.3 nm in width. Then, a W tip was moved leftward and pressed the particle to a thickness of only 7.7 nm in Figure S5(b). After that, the W tip was moved rightward and thus stretched the particle due to the van der Waals forces. When the particle was stretched, some fresh surfaces were also formed in Figure 2(d). Then, similar with the results in Figure S4, a new PbO_x_ layer grew on the clean surfaces since the whole system is under electron beams, as shown in Figure 2(e). Finally, a PbO_x_ layer again covered the whole Pb particle in Figure 2(f). As a result, the Pb particle grew into an irregular shape with its length increased to 16.7 nm and width decreased to only 10.0 nm as shown in Figure S5(f). Different from the results shown in Figure 1, this particle did not recover its initial shape in Figure 2(c), indicating that surface passivation led to plastic deformation instead of pseudoelastic deformation of Pb particles. Other similar results are shown in Figure S6. In all cases, diffusion-induced liquid-like spontaneous shrinking of Pb particles after stretching was suppressed by surface oxidization, which led to irregular particle shapes after unloading.

Moreover, we also found that surface passivation prevents coalescence of Pb particles (Figures 2(g)–2(i)), in stark contrast with the case in Figures 1(h)–1(j). Note that plastic deformation is observed in both particles after detachment in Figure 2(i).

**Figure 3:**
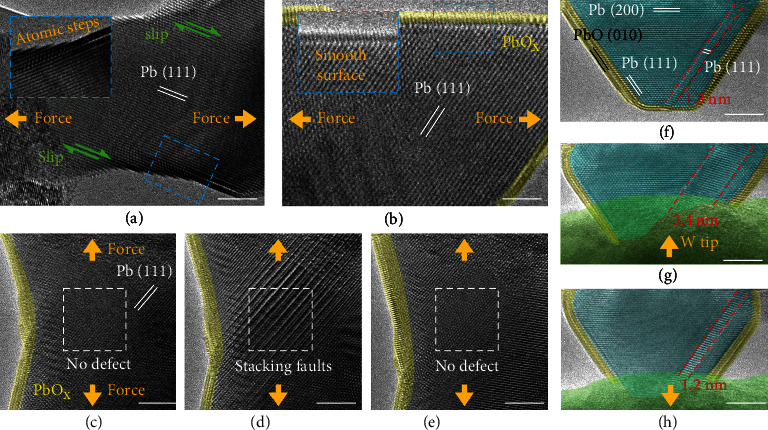
Displacive deformation of Pb particles during *in situ* deformation process. (a and b) Surface contour of pure (a) and passivated (b) Pb particles during stretching process. (c–e) Evolution of stacking faults inside Pb particle during stretching. (f–h) Evolution of twin structure during *in situ* extrusion and stretching. For better view, the PbO layers have been painted yellow. The orange arrows in (g) and (h) indicate the movement direction of W tip. Scale bars: 5 nm.

**Figure 4:**
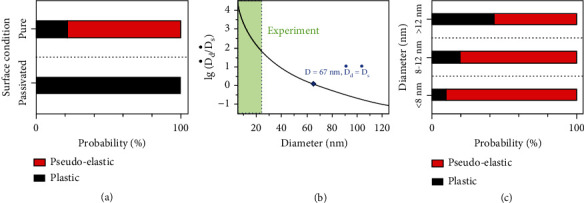
Half-quantitative comparison of plastic and pseudoelastic deformation processes of Pb particles with different surface conditions. (a) Measured probability of plastic and pseudoelastic deformation processes of Pb particles with different surface conditions. (b) Comparison of diffusive and displacive deformation rate of pure Pb particles with different sizes. (c) Measured probability of plastic and pseudoelastic deformation processes of pure Pb particles.

Figures 1 and 2 have shown dominant diffusive pseudoelastic deformation of clean Pb particle and plastic deformation of surface-passivated Pb particle. According to previous studies, the deformation mode of pristine nanometals is mediated by a size-dependent competition between diffusive and displacive processes [18]. In our case, based on the observations in Figure 1 (diffusion) and Figure 3(a) (slip), pure Pb nanoparticles are found to undergo a combination of both deformation modes. To deeply understand the dominant deformation mechanism of Pb nanoparticles and quantify the influence of surface passivation, mechanical tests were conducted on more than 100 Pb particles with different surface conditions. Note that pseudoelasticity can mainly be attributed to the diffusion of surface atoms and thus can be viewed as an indicator of dominant diffusive deformation. That is, the existence or absence of substantial pseudoelasticity is used for differentiate between diffusive-dominant and displacive-dominant deformation modes. A statistic study (Figure 4(a)) reveals that ~78% of the clean Pb particles exhibited dominant diffusive pseudoelastic deformation, while only 22% showed certain degrees of displacive plasticity (such as the slip process in Figure 3(a)). By contrast, all surface-passivated Pb particles demonstrated displacive plastic deformation absent of pseudoelasticity. Such dramatic differences can be understood by considering the size-dependent competition between the diffusive and displacive deformation modes. In the case of pure Pb particles, a half-quantitative analysis similar to a previous study on Ag nanoparticles [18] was performed by comparing the diffusive and displacive deformation rate of pure Pb particles with different sizes during the stretching process, as shown in Figure 4(b) and Figure S7. A threshold particle diameter of 67 nm was derived, below which diffusive deformation overwhelms displacive deformation and vice versa. Such findings rationalize the prevailing of diffusive deformation in Pb nanoparticles with diameters below 25 nm (Figure 4(c)). In the case of surface-passivated Pb particles, however, the surface oxidation layer effectively suppresses the diffusion of Pb atoms at the layer interface and consequently diffusive deformation, making displacive deformation the exclusive dominant deformation mode for all tested Pb particles (Figure S8). These findings highlight the dramatic impact of surface condition on the mechanical behavior and properties of small-volume metals.

Due to the confinement of the surface oxide layer, crystal slips in surface-passivated Pb particles proceed in a different manner compared to those in clean particles. A comparison between Figures 3(a) and 3(b) reveals that the particle shape during plastic deformation keeps much smoother in passivated particles than in clean particles. Due to the lack of work hardening mechanisms, small-volume metals often suffer from plastic instability arising from localized dislocation dynamics or crystal slips (Figure 3(a)). Such localized deformation generates continuously thickening surface steps and thus a rough surface contour in pristine particles [10, 14]. By contrast, in surface-passivated particles, the formation of abrupt changes in the surface contour is prohibited by the surface oxide layer, which promotes uniform deformation by either suppressing localized dislocation slips or activating alternative slip mode such as twinning and stacking fault formation. As shown in Figures 3(c)–3(e), the gradual formation and disappearance of stacking faults by emission and migration of partial dislocations were captured during *in situ* stretching of surface-oxidized Pb particles. And twinning/detwinning processes were more favored in surface-passivated particles (Figures 3(f)–3(h)) than in clean particles. This can be rationalized by the fact that partial dislocation-mediated deformation generates thinner surface steps and thus smoother surfaces compared to full dislocation slip, especially in the case of twinning/detwinning where the particle surface contour is gradually changed by sequential migration of twinning partial dislocations on successive adjacent crystal planes [19, 20]. In addition, surface passivation also increases the stress required for driving plastic deformation. As demonstrated in the half-surface-passivated Pb particle in Figure S9, upon tensile loading, the segment with a clean surface stretched noticeably, while the passivated segment kept stable. These findings offer an effective approach for increasing the mechanical stability of small-volume metals.

With clean surfaces, diffusive deformation process is found dominant in metals with low activation energy barrier for atomic diffusion, for example, Pb, Ag, and Sn [12, 16, 18, 27, 31]. However, in ambient environments, most metals, except a few inert ones such as Au and Pt, are prone to surface oxidation, whose impact on mechanical behavior was often neglected and yet to be clarified. Based on our observations, changes in surface condition can dramatically alter the deformation process and thus the mechanical properties of small-volume metals. As such, we believe that except for common factors such as the temperature and material size, surface condition is another important factor that should be taken into account when investigating mechanical properties of materials. The surface condition effect is expected to be most prominent in active metals with small sizes and good surface diffusivities, such as Cu, Bi, Sn, and Ag.

## 3. Conclusions

In this research, the mechanical deformation process of Pb nanoparticles with different surface conditions was studied by *in situ* experiments inside an aberration-corrected Titan TEM/STEM. It was found that clean and oxidized surfaces, respectively, lead to dominant pseudoelastic behavior and plastic deformation. During these experiments, the deformation process was determined by the probability of surface diffusion of Pb atoms. For Pb particles with clean surfaces, diffusion of Pb surface atoms prevails and leads to pseudoelasticity. When surfaces of Pb particles are covered with PbO_x_, surface diffusion is suppressed and gives way to displacive deformation processes, including dislocations, stacking faults, and twinning. This research opens up new opportunities to tune the mechanical properties of small-volume metals by surface engineering and provides implications for developing new advanced nanodevices in the future.

## 4. Materials and Methods

### 4.1. Synthesis of Pb Particles

The Pb particles are obtained based on *in situ* heat treatment of PX phase PbTiO_3_ nanowires. The PX-PbTiO_3_ nanowires are prepared in a hydrothermal method: firstly, 4 mmol Ti(OC_4_H_9_)_4_ was dissolved in 8 mL of ethanol, and then, the transparent solution was hydrolyzed in 8 mL of deionized H_2_O. Next, 20 mmol KOH, 5.2 mmol Pb(CH_3_COO)_2_^∗^3H_2_O, and 0.050 g polyvinyl alcohol (PVA) were added into the solution and mixed under stirring. The feedstock was transferred to a 50 mL Teflon-lined autoclave and held at 200°C for 3.5 h after adjusted to 40 mL with deionized H_2_O. The obtained products were washed with deionized H_2_O and 10 wt% CH_3_COOH solution repeatedly until the remnant PbO was totally removed. Finally, after dried at 60°C in air, the pure PX phase nanowires were obtained.

Then, to prepare the Pb particles, firstly the PX-PbTiO_3_ nanowires were dissolved in ethanol by ultrasonic dispersion. Then, the suspension was dropped onto a half Cu grid and dried in air for 5 min. The Cu grid was put on a TEM-thermal holder (Gatan 628 Single Tilt Heating Holder) and heated at 350°C at the highest heating rate under the radiation of electron beams with the beam density of about 4^∗^10^4^ A/m^2^.

The surface condition of Pb particles is tuned by the amount of electron beam irradiation time. As shown in our previous research [32], the pristine PX-PbTiO_3_ structure was destroyed by electron beam irradiation. Pb atoms diffuse to the surface of the nanowires and form pure Pb particles, while most of the O atoms (or O_2_) escape into the environment (only a small fraction of oxygen is adsorbed on the nanowire surface). Within a time period of ~10 minutes, the as-formed Pb particles can maintain clean surfaces since the amount of adsorbed oxygen is not enough to generate an oxide layer. During prolonged irradiation, however, the concentration of absorbed surface oxygen continuously increases, leading to the formation of passivated Pb particles with an intact surface oxide layer.

All the TEM characterization and the *in situ* experiments were carried out inside the aberration-corrected TEM (FEI Titan 80-300).

### 4.2. *In Situ* Mechanical Tests

As for the *in situ* mechanical tests, after the Pb particles were prepared on the half Cu grid, the grid was fast transferred to a TEM-scanning tunneling microscopic holder by glued onto a gold wire with conductive epoxy. Then the *in situ* mechanical experiments on the Pb particles were conducted using some tungsten tips as prepared by electrochemically etching: 2 mol/L NaOH was used for the etching and the etching voltage was 2.5 V with a compliant current of 20 mA. All W tips were cleaned by plasma cleaning for 5 minutes for the *in situ* TEM experiments. The *in situ* compression and stretching process of the Pb particles were conducted at a speed approximate 0.3 nm/s.

## Data Availability

All data are available in the manuscript, supplementary materials, or from the author.

## Supplementary Materials

Supplementary Materials Half-quantificational comparison of plastic and pseudoelastic deformation processes of pure Pb particles. Figure S1: in situ oxidization of Pb particles. Figure S2: characterization of Pb particle covered by multilayers of PbO. Figure S3: liquid-like pseudoelasticity deformation of pure Pb particle. Figure S4: in situ repairing of PbO layers. Figure S5: plastic deformation of surface-passivated Pb particle. Figure S6: behavior of clean and surface-passivated Pb particles after the stretching process by W tip. Figure S7: illustration of slip and diffusion process of pure Pb particles. Figure S8: statistics of plastic and pseudoelastic deformation of surface-passivated Pb particles with different diameters. Figure S9: mechanic deformation of Pb particle with part of surface covered with PbO layers. Table S1: interplanar spacing of PbO and Pb (Supplementary Materials).
