# Supplementary material for: Surface-Condition-Dependent Deformation Mechanisms in Lead Nanocrystals
Source: Research (Wash D C). 2022 Jul 27;2022:9834636. doi: 10.34133/2022/9834636 (PMC9362692; doi:10.34133/2022/9834636)
Supplement: Supplementary Materials — Half-quantificational comparison of plastic and pseudoelastic deformation processes of pure Pb particles. Figure S1: in situ oxidization of Pb particles. Figure S2: characterization of Pb particle covered by multilayers of PbO. Figure S3: liquid-like pseudoelasticity deformation of pure Pb particle. Figure S4: in situ repairing of PbO layers. Figure S5: plastic deformation of surface-passivated Pb particle. Figure S6: behavior of clean and surface-passivated Pb particles after the stretching process by W tip. Figure S7: illustration of slip and diffusion process of pure Pb particles. Figure S8: statistics of plastic and pseudoelastic deformation of surface-passivated Pb particles with different diameters. Figure S9: mechanic deformation of Pb particle with part of surface covered with PbO layers. Table S1: interplanar spacing of PbO and Pb (Supplementary Materials). [file 9834636.f1.doc]

Supplementary Materials

**Surface-Condition-Dependent Deformation Mechanisms in Lead Nano-Crystals**

**Hongtao Zhang^1^†, Wen Wang^1^†, Jun Sun^1^, Li Zhong^1^*, Longbing He^1^*, Litao Sun^1, 2^***

*^1^SEU-FEI Nano-Pico Center, Key Laboratory of MEMS of Ministry of Education, Southeast University, Nanjing 210096, China.*

*^2^Center for Advanced Materials and Manufacture, Southeast University-Monash University Joint Research Institute, Suzhou, 215123, China.*

Correspondence should be addressed to Li Zhong; [lizhong@seu.edu.cn](mailto:lizhong@seu.edu.cn); Longbing He; [helongbing@seu.edu.cn](mailto:helongbing@seu.edu.cn) and Litao Sun;  [slt@seu.edu.cn](mailto:%20slt@seu.edu.cn)

**†** These authors contributed equally to this work.

1. **Half-quantificational comparison of plastic and pseudo-elastic deformation processes of pure Pb particles.**

According to the model of Ag nano-particle (reference 18), the theoretical deformation model of pure Pb particles is shown in FigureS7. The thinning rate of Pb by slip should be:

#

$$\frac{dD_{\mathrm{slip}}}{\mathrm{dt}}=\frac{\dot{\varepsilon}\mathrm{LDsin} \alpha}{D\cos\alpha-\varepsilon_{0}L\sin\alpha}$$

Meanwhile thinning rate of Pb by diffusion should be:

#

$$\frac{dD_{\mathrm{diffuse}}}{\mathrm{dt}}=\frac{{\Omega\delta}_{s}D_{s}}{k_{B}T}(\frac{4\gamma}{L^{2}D}+\frac{15E{\varepsilon_{0}}^{2}}{16L^{2}})$$

Here, $\dot{\varepsilon}$ is deformation rate, L is length of Pb particle, D is diameter, α is the projected angle with respect to the <112> loading direction of the {111}<110> slip, ε0 is elastic limit, Ω is atomic volume, δs is atomic diameter, kB is Boltzmann constant, T is temperature, γ is surface energy, and E is Young modulus.

1. **Supplementary Figures**


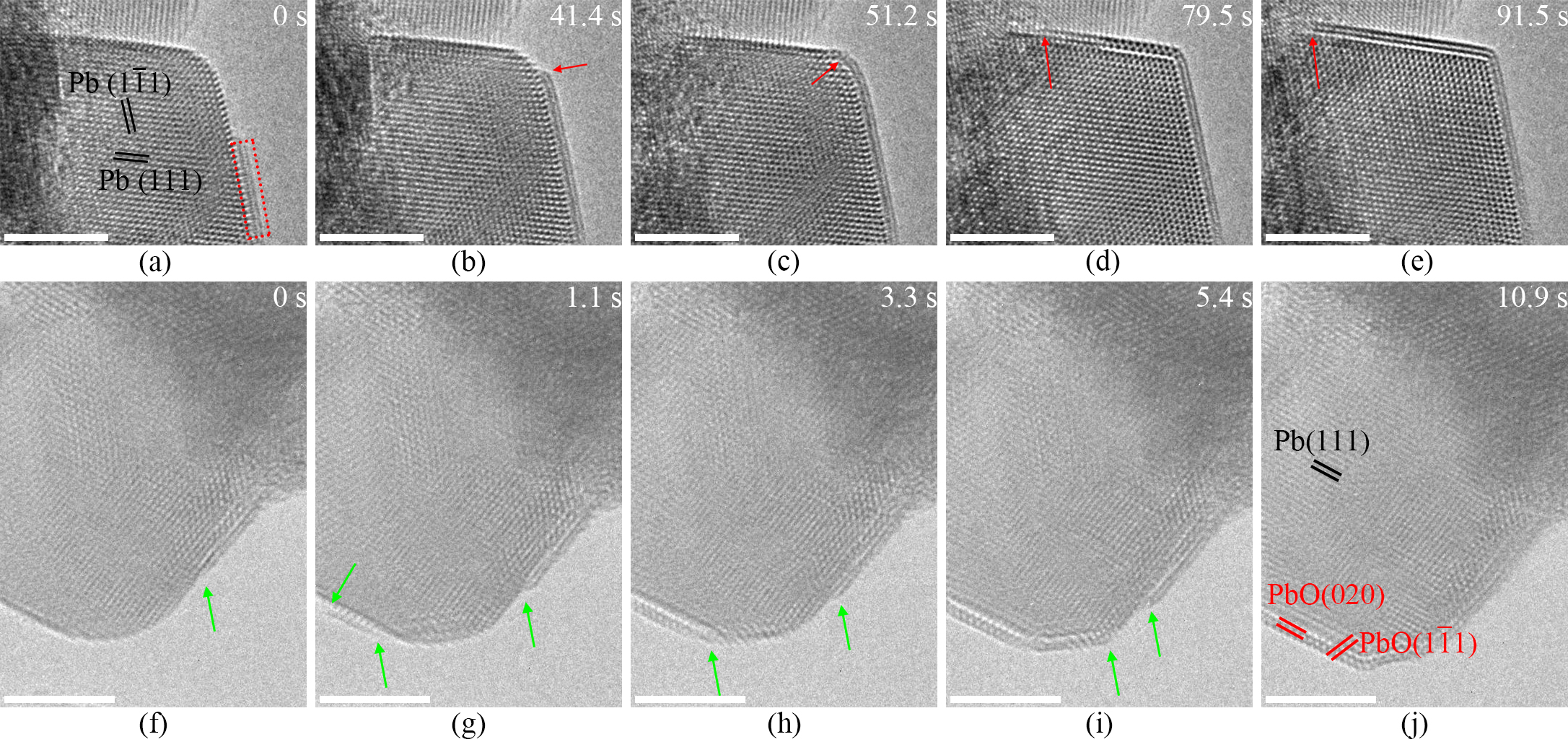


**Figure S1: *In situ* oxidization of Pb particles. (a)-(e)** *In situ* growth of PbO layers along Pb particle surface. The red arrows indicate the head front of the PbO layers. **(f)-(j)** *In situ* formation of whole PbO layers on particle surface. The green arrows indicate the head fronts of the PbO layers. Scale bars: 5 nm.


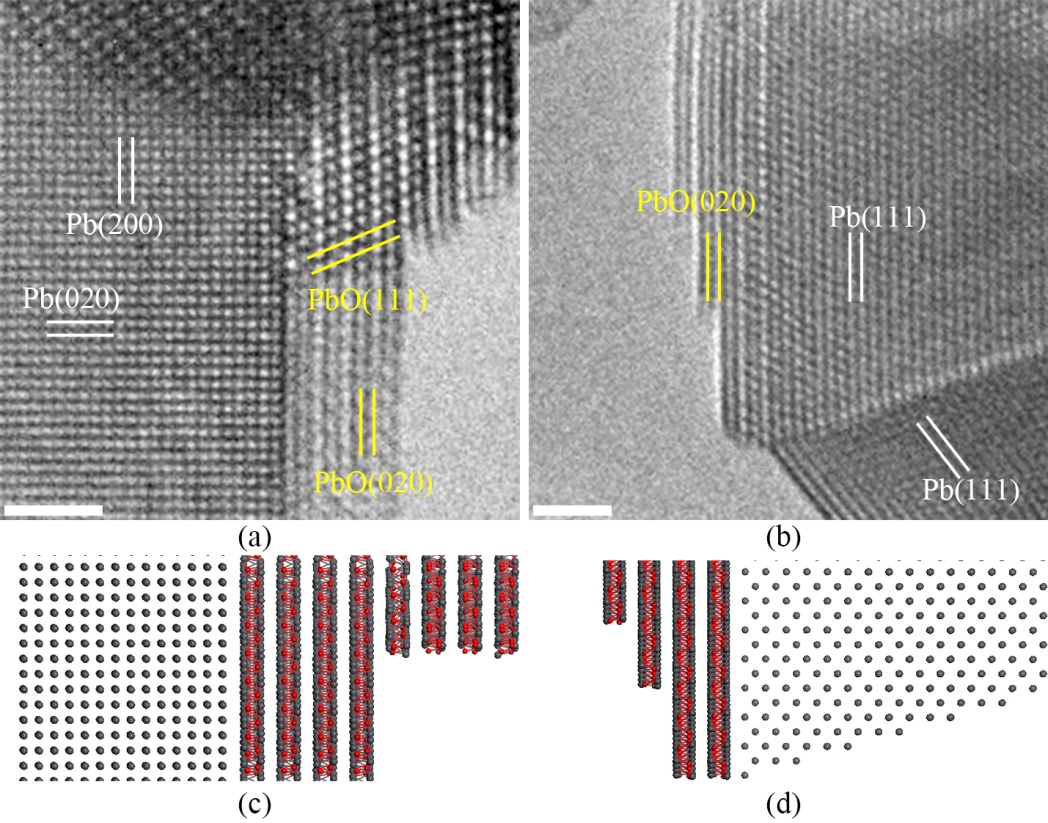


**Figure S2: Characterization of Pb particle covered by multi-layers of PbO. (a)-(b)** Experimental TEM images of surface-oxidized Pb particles. **(c)-(d)** Models of Pb particles based on (a)-(b). Scale bars: 2 nm.


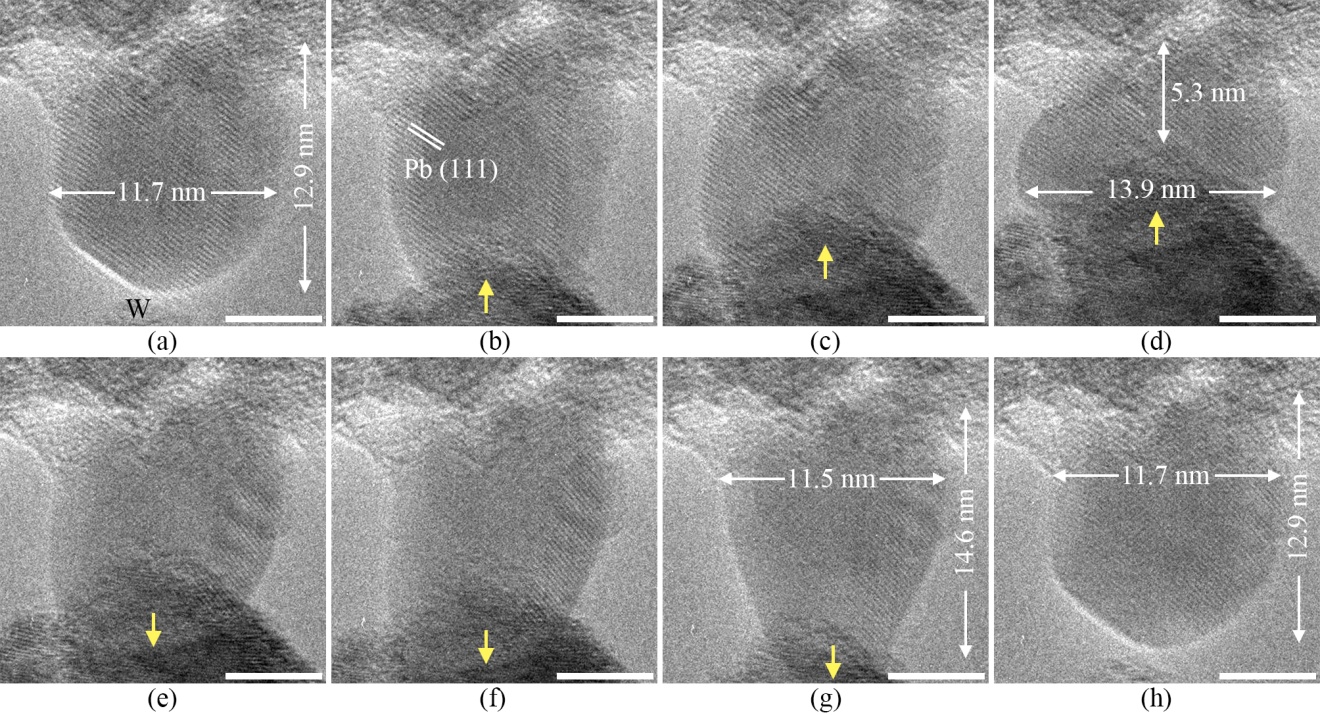


**Figure S3:** **Liquid-like pseudoelasticity deformation of pure Pb particle. (a)** Initial morphology of Pb particle before deformation. **(b)-(d)** Shape evolution of the Pb particle during *in situ* extrusion. **(e)-(g)** Shape evolution of the Pb particle during *in situ* stretching. **(h)** Final morphology of the Pb particle after deformation. Scale bars: 5 nm.

**
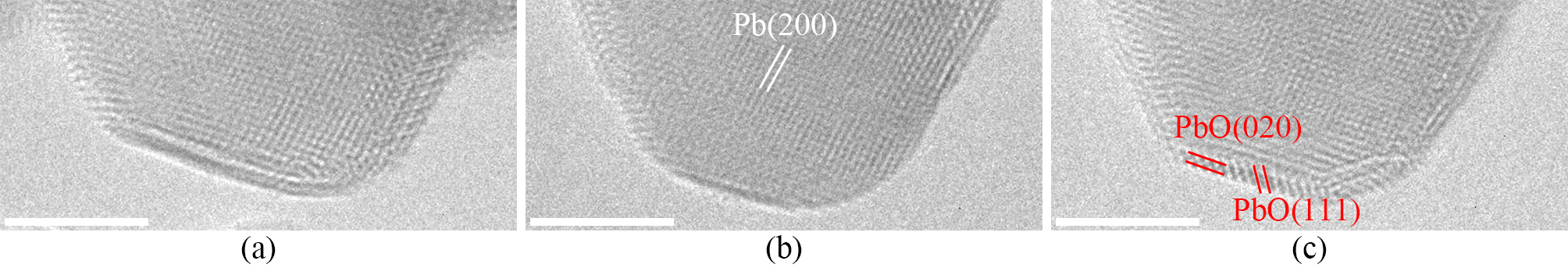
**

**Figure S4: *In situ* repairing of PbO layers. (a)** Initial morphology of PbO-covered Pb particle. **(b)** Morphology of the particle after the PbO layers was destroyed by W tip. **(c)** Final morphology of the Pb particle after *in situ* repair of PbO layers. Scale bars: 5 nm.


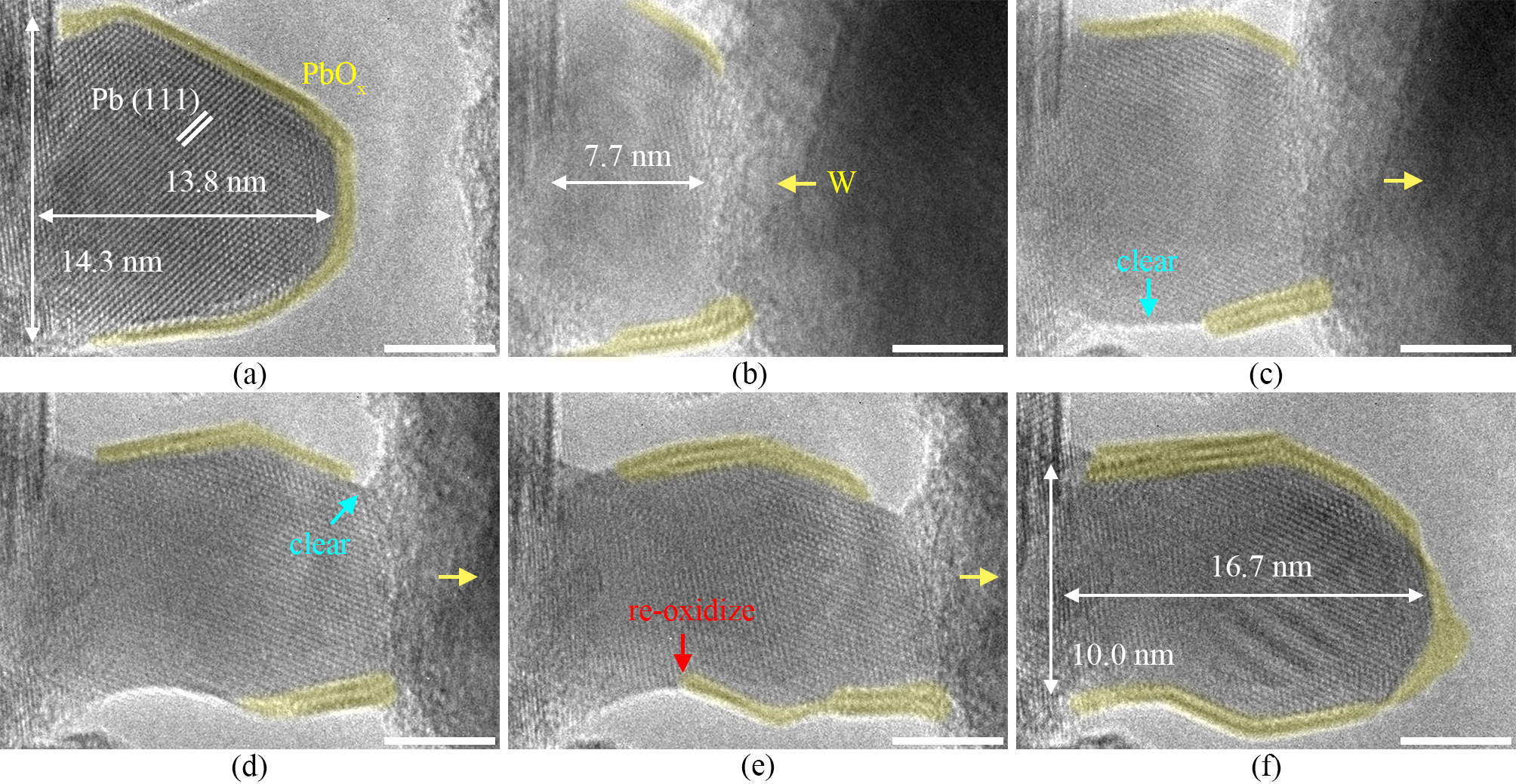


**Figure S5: Plastic deformation of surface-passivated Pb particle. (a)** Initial morphology of Pb particle before deformation. **(b)** Shape of the Pb particle during *in situ* extrusion. **(c)-(e)** Shape evolution of the Pb particle during *in situ* stretching. **(f)** Final morphology of the Pb particle after deformation. Scale bars: 5 nm.


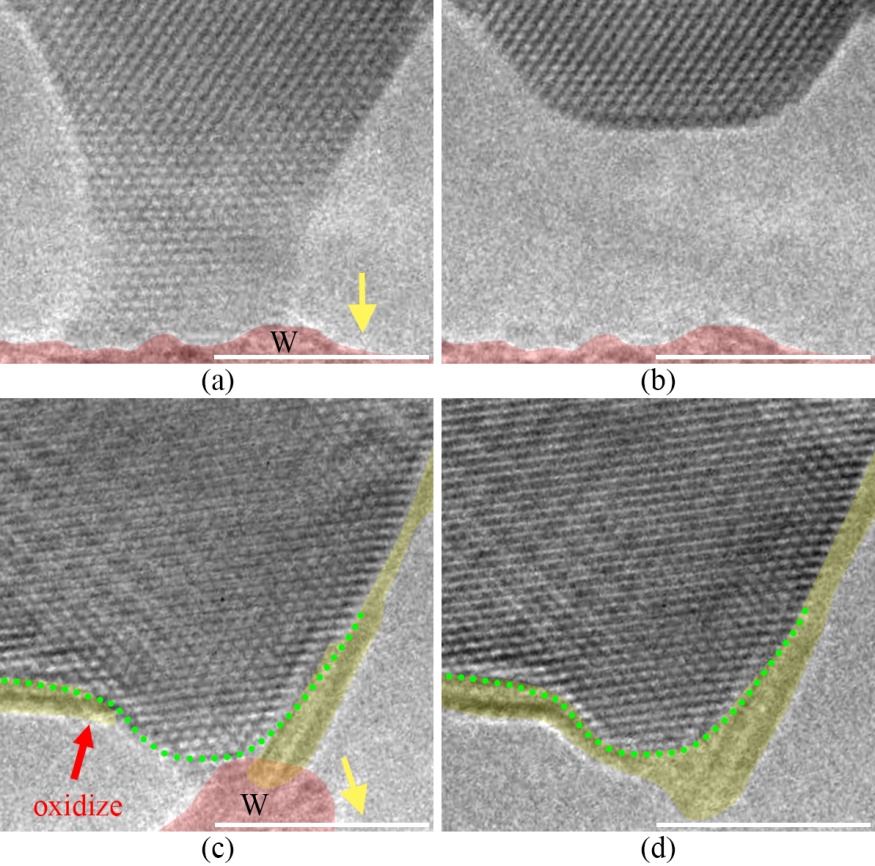


**Fig S6: Behavior of clean and surface-passivated Pb particles after the stretching process by W tip. (a)-(b)** Morphology of clean Pb particle (a) at the moment lost contact with the W tip and (b) after the stretching. **(c)-(d)** Morphology of PbO-covered Pb particle (c) at the moment lost contact with the W tip and (d) after the stretching. For better view, PbO_x_ layers have been painted yellow. The green dotted lines show the shape of Pb. The red arrow in (c) shows the growth of the PbO layer. The yellow arrows indicate the movement direction of W tip. Scale bars: 5 nm.


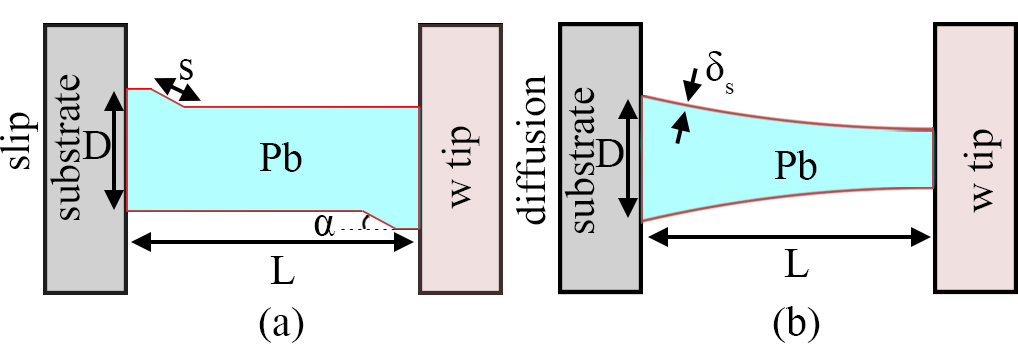


**Fig S7: Illustration of slip (a) and diffusion (b) process of pure Pb particles.**


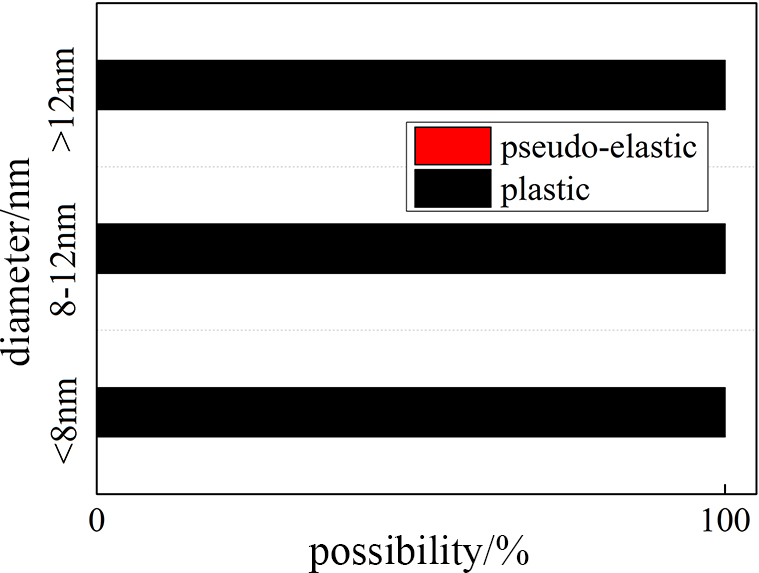


**Fig S8: Statistics of plastic and pseudo-elastic deformation of surface-passivated Pb particles with different diameters.**


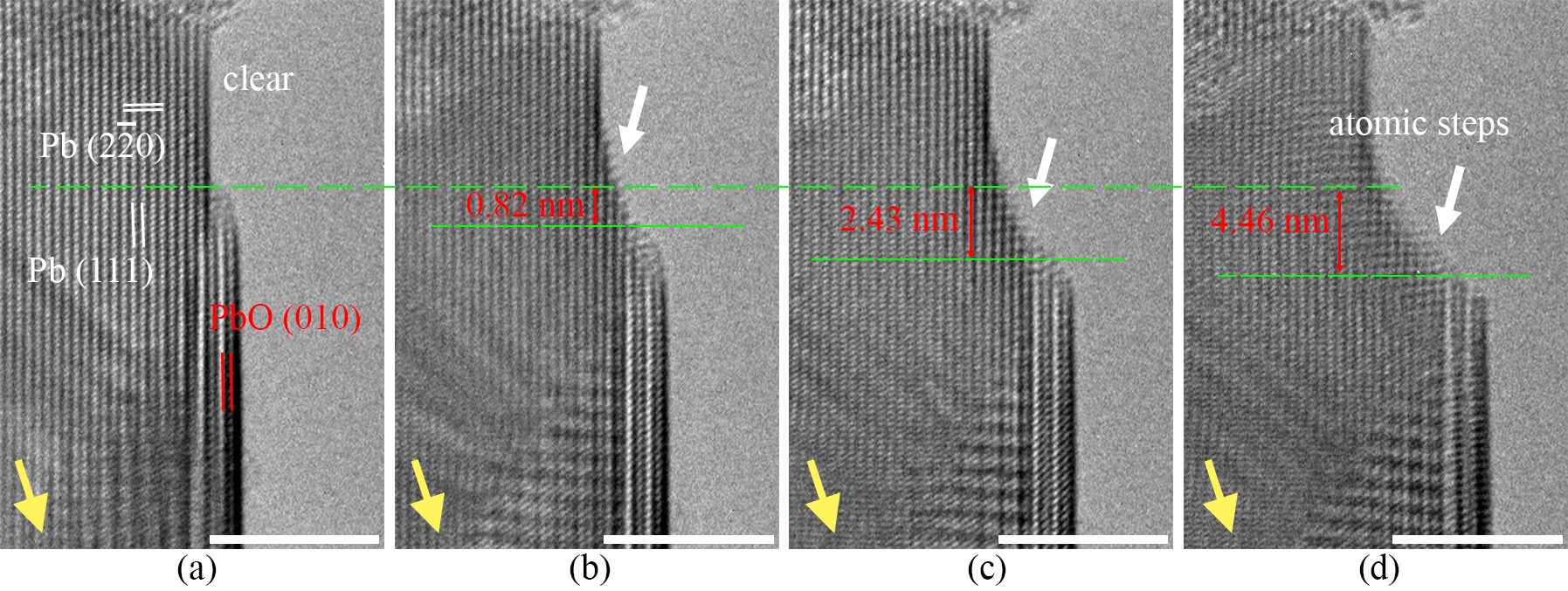


**Fig S9: Mechanic deformation of Pb particle with part of surface covered with PbO layers. (a)** Initial morphology of the Pb particle with bottom part covered with PbO. **(b)-(d)** The *in-situ* stretching process of the particle by W tip. The green dotted lines show the upper edge of the PbO layers. The white arrows indicate the Pb atomic steps formed during the stretching. The yellow arrows indicate the movement direction of W tip. Scale bars: 5 nm.

**Table S1:** **Inter-planar spacing of PbO and Pb**

| Pb(Cubic, Fm-3m)  Cell: 4.951Å*4.951Å *4.951Å <90°*90°*90°> | | PbO(Orthorhombic, Pcam)  Cell: 5.490Å*5.892Å *4.752Å <90°*90°*90°> | |
| --- | --- | --- | --- |
| lattice plane | Inter-planar spacing/Å | lattice plane | Inter-planar spacing/Å |
| (100) | 4.951 | (110) | 4.017 |
| (110) | 3.501 | (111) | 3.068 |
| (111) | 2.855 | (020) | 2.946 |
| (200) | 2.475 | (200) | 2.745 |
| (220) | 1.750 | (201) | 2.377 |
